# Scientific communication

**DOI:** 10.4103/0019-5413.30516

**Published:** 2007

**Authors:** Anil K. Jain

**Affiliations:** Professor of Orthopedics, University College of Medical Sciences and Guru Teg Bahadur Hospital, Delhi - 95, India. E-mail: editor@ijoonline.com

The objective of the Indian Journal of Orthopaedics is to provide high-quality peer reviewed articles in the form of original research. Scientific writing is a demanding creative process and not merely an act. The process of writing changes thoughts and thoughts change writing. The quality of a report depends on the quality of thought in the design and the rigor of the conduct of the research. The brevity and focus on the core issues are critical to the effectiveness of the report. Scientific communication is an important tool for the growth of science and to improve the quality of day-to-day clinical practice. Scientific analysis in clinical practice improves skills and clinical outcome. The scientific analytic approach needs to be inculcated in our practice. Each and every patient treated needs to be evaluated in terms of scientific parameters. “What was done was perfect or not”, “could I have done better” are the questions every surgeon asks oneself every day. These questions stimulate outcome evaluation. The outcome evaluation, if scientific, not only improves the clinical performance, but also makes it more predictive and prevents complications.

Scientific writing is a tool by which the knowledge that a person has gained by scientific evaluation of a patient becomes helpful to others. Scientific writing has to be followed in a systemic manner so that it is crisp and communicates with the reader well. Scientific writing is an art that comes by practice only. The analysis of all write-ups over the years by one person shows a trend that reflects his growing maturity in writing skills. Sir Grahm Apley once wrote, “writing is like having a baby; the gestation period is long and the labor is painful, but in the end you have something to show for it”.^1^ The write-up should be crisp, clear and informative. The author may have various thoughts in mind and hence the write-up may be too abbreviated or too lengthy. One should always keep the average reader in mind, who is not well versed with the subject but inquisitive to know the subject.

Before deciding to undertake research and to write, the author has to scan the literature thoroughly. The introduction should include the shortcomings in the present knowledge that prompted the author to conduct the study. Materials and Methods should give enough information for others to be able to repeat your work. The author should specifically describe the data with the help of a statistician if needed. It should be clear as to how the results are assessed and presented. The description of the data should be well understood with the help of a suitable table and pie chart. Organize the conclusions and discuss the significance of your findings with the available literature. The simpler conclusions are more memorable. Whatever has been written is to be assembled as a manuscript in a logical sequence. Every article needs polishing. Once the article is written, leave it in a drawer for two to three weeks.[1] The author may find needless repetition and irrelevancies in another reading. Give the manuscript finally to one of your colleagues to read, who is not a part of the study. His suggestions are most important to make the manuscript clear and informative as he is acting as a reader. This gives a crisp version of the manuscript. In conclusion the authors must provide complete, accurate data and reader will make a decision regarding a patient on the basis of this data.

The editor's responsibility to authors is to help them to present their materials clearly, concisely and accurately.[2] The editor has to ensure that only valid accurate articles that benefit the readers and are worth their time should be published. Only those articles that enhance patient care deserve a place in the literature. The authors and editors have to work in perfect coordination, without losing the focus of achieving upgradation and continuum of the scientific growth in the best interest of patients in particular and society at large. The editing of a manuscript involves correction of the data without altering the meaning of the manuscript and not to express disapproval to the authors. It involves a lot of exchange between the author and the editor. Together the editor and authors should determine what the readers want to know and how to supply this information. The editors ask questions that he/she thinks will be asked by the readers and the answers will result in a comprehensive text. The authors must remember that their ultimate goal is to satisfy the reader. There is a permanence to the material published in the journal and the editor is attempting to save them from the embarrassment that would result from inaccurate publication.^2^ An improvement in communication skills makes the author better communicative with the patient family and society. The practice of medicine depends on accuracy in the scientific literature. The ultimate goal of the editorial process is the presentation of accurate and valid data to the reader.
